# Aponeurosis discission, a low-detergent method for tissue-engineered acellular ligament scaffolds

**DOI:** 10.1007/s10856-022-06661-8

**Published:** 2022-05-04

**Authors:** Sheng-yuan Zhou, Bo Yuan, Wen-mao Huang, Xiong-sheng Chen, Lian-shun Jia

**Affiliations:** Spine Center, Department of Orthopedics, Shanghai Changzheng Hospital, Naval Medical University (Second Military Medical University), Shanghai, 200003 China

## Abstract

**Abstract:**

Detergent treatment is the most commonly used method for the decellularization of ligaments and tendon grafts. However, it is well recognized that detergent treatment can also adversely affect the extracellular matrix. This study found that discission into the aponeurosis layer of the patellar tendon (PT) before decellularization is conducive to extracting cells from the PT using a low quantity of detergent in a short time period. The acellular aponeurosis discission ligament (AADL) retains its native collagen fibril structure and mechanical properties. Moreover, the PT retained cell and tissue compatibility in vitro and in vivo. After implantation into a defective allogeneic PT, we found that the AADL healed well in the host, and its collagen structure exhibited gradual improvement 12 months after implantation with satisfactory reconstruction.

**Impact:**

The aponeurosis of tendons/ligaments is the main barrier to achieving complete decellularization, and it thus prevents complete recellularization for applications in tissue engineering. Aponeurosis can obstruct the removal of cell components. We found that excising the aponeurosis before decellularization allows for the removal of cellular components with a reduced amount of detergent, thus improving the biological properties of the acellular ligament. To the best of our knowledge, no similar studies have been performed.

**Figure:**
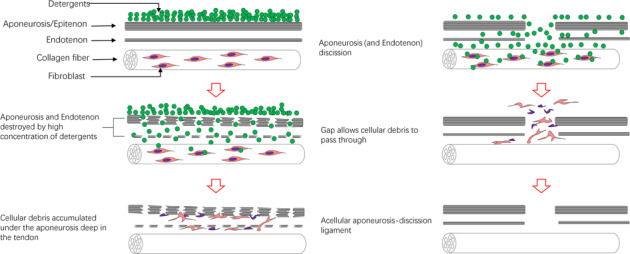
Graphical abstract

## Introduction

Current tissue-engineering ligament scaffolds are derived from a variety of substances, such as silk [1], natural collagen [2], polyglycolic acid/polylactic acid and their copolymer (PLGA/PLLA) fibers [3–5], and ligament/tendon extracellular matrices [6–9]. Acellular ligament scaffolds have the properties of the native ligament extracellular matrix (ECM). Its immunogenicity is reduced by the extraction of cell components [10–13] and has only minimal antigenicity preserved due to type-I collagen [14, 15]. Therefore, acellular ligament scaffolds are expected to be the ideal tissue-engineered ligament scaffold.

Existing acellular ligament scaffold studies utilize pig anterior cruciate ligaments [6], pig or rabbit patellar tendons [7, 8], and chicken toe deep tendons [9]. Decellularization protocols are often combined with physical, enzymatic, and chemical treatments. The application of chemical detergent is the primary step to achieving decellularization [16]. The physical and enzymatic decellularization of ligaments/tendons do not differ much; however, detergents used for their decellularization effects have been reported to exhibit different properties in different species, tissues, and ligament/tendon sizes [6–10, 16–18]. Commonly used detergents for ligament decellularization contain sodium dodecyl sulfate, tri (n-butyl) phosphate, and Triton X-100, all of which may cause damage to collagen and the glycosaminoglycans of the ECM [6, 17]. Therefore, reducing the dose of the detergent may protect the biological characteristics of the ligament ECM.

**Fig. 1:**
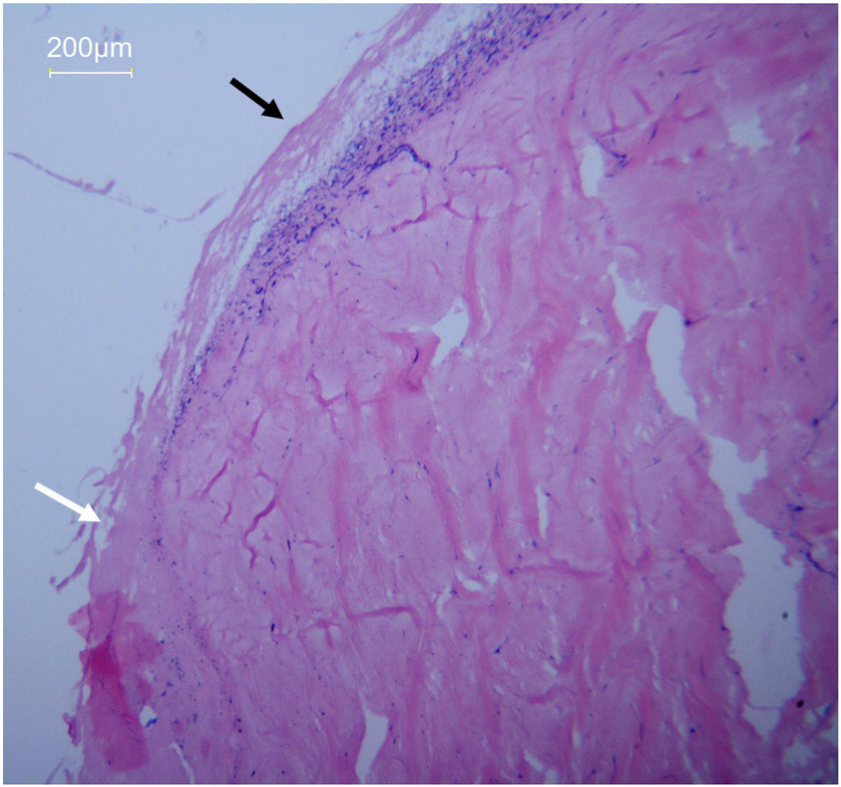
Hematoxylin and eosin (H&E) staining of the rabbit Achilles tendon treated with hypotonic Tris buffer without chemical detergents. Major nuclear components accumulated under the intact aponeurosis (black arrow), and a small amount of residual nuclear components were visible at the edge of the incomplete aponeurosis (white arrow) (magnification ×100)

In our previous study on the decellularization of the rabbit Achilles tendon, we found that, when treated with hypotonic Tris buffer for 48 h in a 150-rpm shaker without chemical detergents, a small quantity of residual cells accumulated under the aponeurosis deep in the tendon. However, in the part of the tendon without an intact, covered aponeurosis, cells were completely removed as determined by hematoxylin and eosin (H&E) staining (Fig. 1). This indicates that the aponeurosis could obstruct the removal of cell components. Therefore, we expected that excising the aponeurosis before decellularization would help to remove cellular components with a reduced amount of detergent, thus improving the biological properties of the acellular ligament.

## Materials and methods

### Harvest of patella tendons

**Fig. 2:**
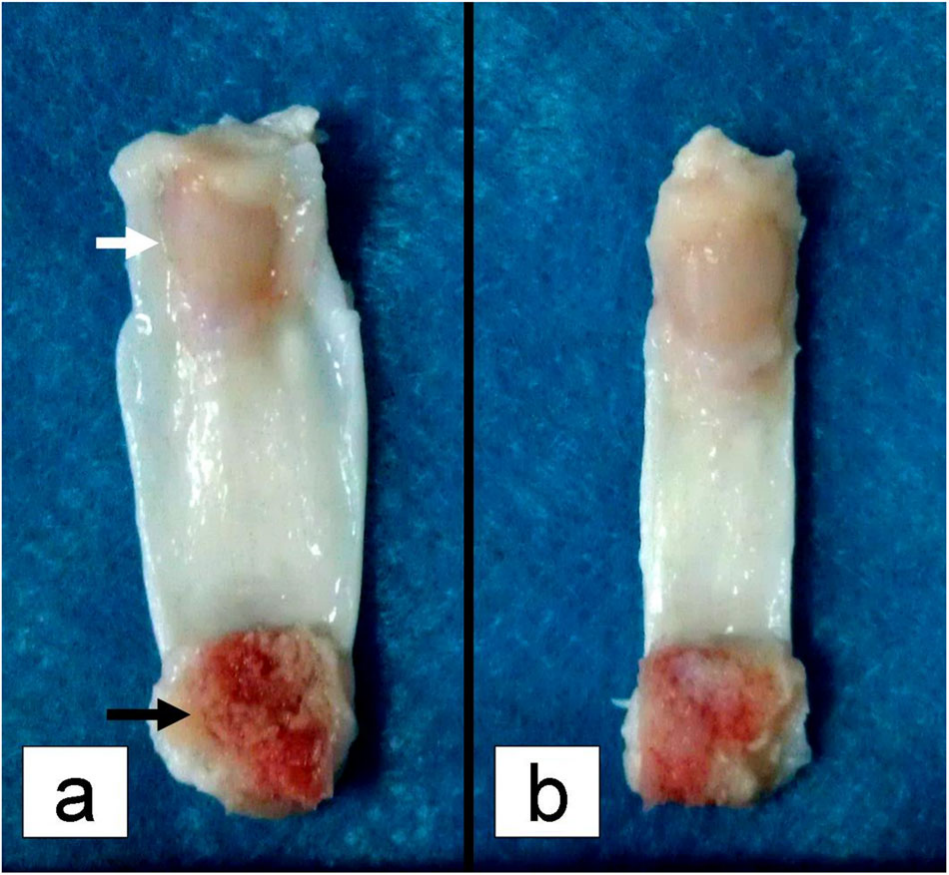
Patellar tendon (PT) aponeurosis preparation prior to decellularization. **a** The patella (white arrow)-PT-tibia (black arrow) complex is retained with intact aponeurosis; **b** aponeuroses were excised on both sides of the PT

The patella tendons (PT) of New Zealand white rabbits (2.5–3.0 kg, 6-month-old male rabbits) obtained from the Second Military Medical University Experimental Animal Center in China were used in this study. The animal husbandry, experiments, and sacrifice were performed according to the animal ethics standards. Induced euthanasia was applied by slow injection of excessive sodium pentobarbital. The patella-PT (12 mm × 8 mm × 1 mm)-tibia complexes (Fig. 2a) were harvested using aseptic techniques. The synovial soft tissue around them was cleared without disrupting the integrity of the PT aponeurosis and then placed into sterile phosphate buffered saline (PBS) for future use. All procedures complied with the ethical use protocols of the Second Military Medical University Experimental Animal Center.

### Preparation of PT aponeuroses prior to decellularization

The aponeuroses of group 1 were excised on both sides of the PT to expose the inner collagen fibers (Fig. 2b). The specimens of group 2 contained intact PT aponeurosis prior to decellularization, and those of group 3 contained fresh PT (FPT) and were used as the control group.

### Decellularization method

The decellularization steps performed were as follows: specimens were (1) immersed into a 10-mM hypotonic Tris buffer for 24 h, (2) transferred to a 50-mM Tris buffer containing a high concentration of saline (1.5 M KCl) for 24 h, (3) treated with 0.05% trypsin for 5 h, (4) washed with PBS and 1% Triton X-100 for 48 h, and (5) washed with PBS for 72 h with the PBS changed every 24 h. All decellularization steps were performed with a serine protease inhibitor (5% phenylmethylsulfonylfluoride in PBS, 0.35 ml/l), a metalloprotease inhibitor (5 mM ethylenediaminetetraacetic acid (EDTA)) and 5 ml/l penicillin solution (10,000 U/ml/10,000 mg/ml) at 37 °C with shaking (100 rpm).

### Histology and scanning electron microscopy analysis

Mid-substance portions of the specimens were obtained and placed in 10% formalin for 8 h at room temperature, dehydrated, embedded in paraffin, microtomed into 5.0-μm thick slices (longitudinal and cross sections), mounted onto slides, and stained with H&E (Sigma). To analyze the nuclear components and collagen structure, a microscope was used to image the sections (NIKON E-600).

After washing with double distilled water, the 10% formalin-fixed specimens were freeze-dried (VirTis BENCHTOP), cut into 1 mm × 1 mm × 1 mm sections, sprayed with a conductive coating to coat the surface, and scanned on an electron microscope (JSM-6700F) [19]. Images were examined for residual cell debris and microstructural changes in the collagen fibrils.

### Determination of DNA and collagen content

Freeze-dried specimens from groups 1, 2, and 3 (dry weight of 60 mg) were recorded. DNA contents were extracted using a tissue genomic DNA extraction kit (TIANamp Genomic DNA Kit; TIANGEN Co., Germany). The DNA concentration in the resulting preparation was used to calculate the total DNA content at an absorbance of 280 nm using a spectrophotometer (Thermo Spectronic, VARIOSKAN) and was then normalized to the initial dry weight of the sample.

Hydroxyproline contents were estimated using a hydroxyproline test kit (Nanjing Jiancheng Bioengineering Institute, China) and measured at an absorbance of 557 nm using a spectrophotometer (Thermo Spectronic, VARIOSKAN). The amount of collagen per dry weight of the sample was determined assuming 13.4% hydroxyproline content in collagen.

### Mechanical testing

**Fig. 3:**
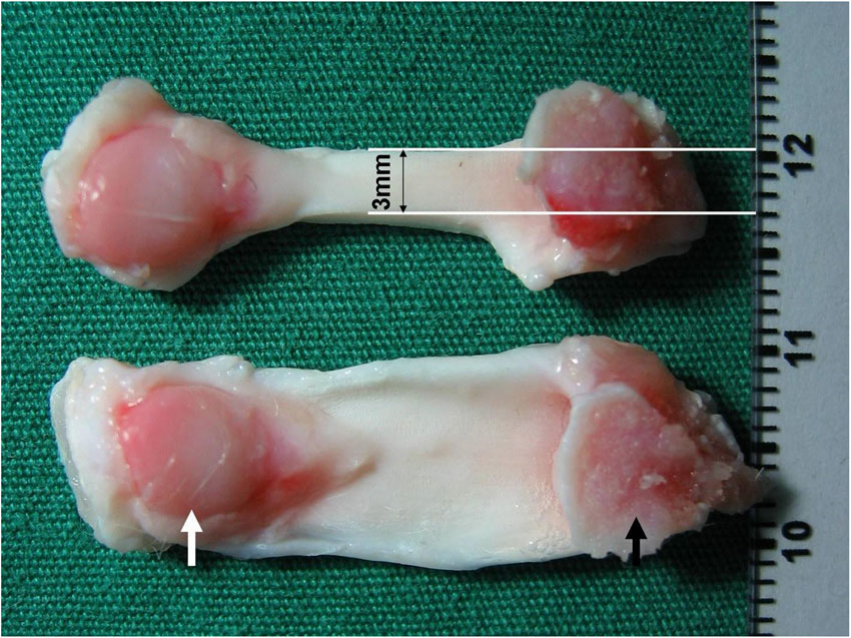
Patellar tendon (PT) aponeurosis preparation before mechanical testing. The PT force area of the patella (white arrow)-PT-tibia (black arrow) complex cut into 3-mm-wide sections

Since the maximum load of a complete rabbit PT is more than 300 N and patella fracture often occurs prior to ligament rupture during mechanical tests, we cut the force area of specimens into 3-mm wide sections (Fig. 3) and used a maximum load of ~200 N to obtain effective biomechanics data of PTs. This load would not lead to patella fracture before ligament rupture. All specimens were stored in PBS at room temperature for 4 h before testing. Their ends, the patella and tibia, were wrapped with PBS-soaked gauze and placed into the grips of a uniaxial load frame (Instron 5542, Needham, MA) to test the maximum load and elastic modulus. Specimens were preloaded at 1 N and loaded at a rate of 15 mm/min, and all PTs were moist with PBS during testing. The measured data for ligament force area rupture without fracture of the patella or tibia were then validated.

### In vitro cytocompatibility of the acellular aponeurosis-discission ligament (AADL)

#### Coculture of cells and the AADL

We cut 50-μm thick slices of AADL with a frozen microtome (LEICA) and washed and preserved them with sterile PBS; further, we packaged these into 15 ml sterile centrifuge tubes and then disinfected them with γ-rays (16 K Gray). The sterile slices were cut into ~10 mm × 10 mm sections and transferred to individual wells (*n* = 6) of a 96-well plate. The wells were coated with collagen (from calf skin, C8919, 10 μg/cm^2^; Sigma) before use and sterilized by overnight exposure to UV light. Furthermore, 100 μl of rabbit PT fibroblast suspension (1 × 10^4^ cells, passage 3, in 10% FBS-DMEM media) tagged with green fluorescent protein (GFP) was dropped onto the surface of the AADL slices and collagen-coated wells and incubated at 37 °C with 5% CO_2_ for 72 h.

#### Colorimetric 3-(4,5-dimethylthiazol-2-yl)-2,5-diphenyltetrazolium bromide (MTT) assay

Cell proliferation was measured by the MTT dye-reduction assay. Briefly, cells were incubated for 24, 48, and 72 h, and coculture specimens were incubated for 4 h at 37 °C with 10 μl MTT solution (5 mg/ml). After removal of the media containing MTT, 100 μl dimethyl sulfoxide was added, and the plates were shaken at room temperature for 10 min. The absorbance was measured at 540 nm using a 96-well plate spectrophotometer.

#### Observation of cells on the AADL

After 24 and 72 h, cocultures and fibroblasts were directly observed on the AADL surface using a fluorescence microscope (NIKON E-600).

At 72 h, the cocultures were fixed with 4% paraformaldehyde for 4 h, rinsed with distilled water, and then lyophilized for electron microscopy examination.

### In vivo cytocompatibility and histocompatibility of the AADL

**Fig. 4:**
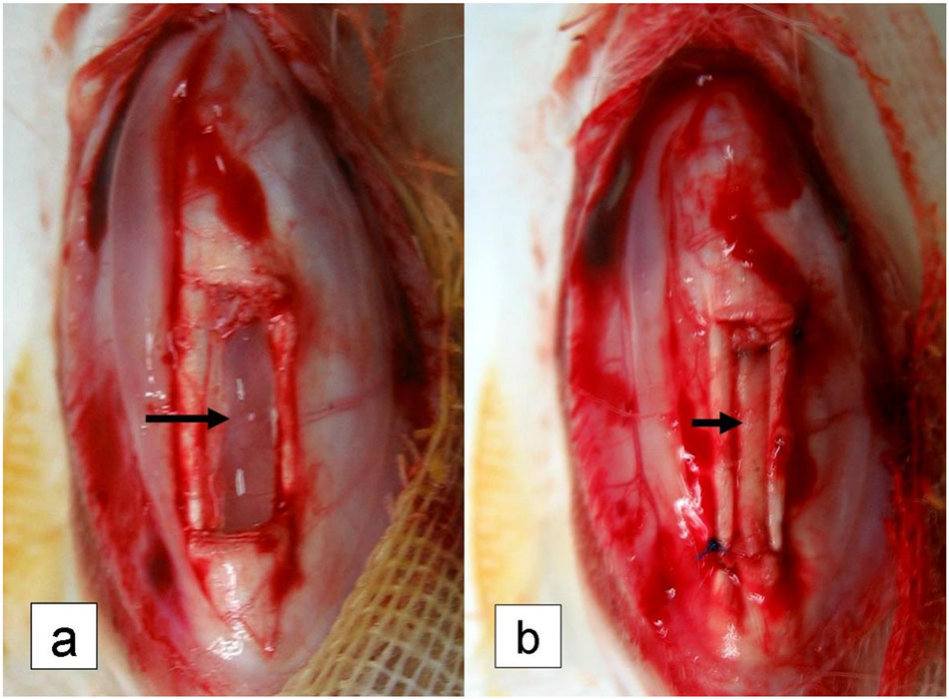
Patellar tendon (PT) defect model establishment and acellular aponeurosis-discission ligament (AADL) implantation. **a** PT defect model (black arrow); **b** AADL implanted into the PT defect (black arrow)

The center of the PT of New Zealand white rabbits, which was ~3 mm × 10 mm × 10 mm in size, was incised to create a defect. The γ-ray (16 K Gray)-disinfected AADL was then implanted and fixed into the PT defect by using a 3–0 nylon suture line for surgery (Fig. 4). Specimens were harvested at 3 weeks, 6 months, and 12 months after implantation and grossly and histologically examined for signs of healing, inflammation/immune response, host cell infiltration, and modification of the AADL structure.

### Statistical analysis

The data from the DNA content, collagen content, tensile analyses, and MTT assay are expressed as the mean values ± standard deviation. The data were subjected to one-way analysis of variance. Statistical significance was determined by an LSD *t*-test, where *p* values ≤ 0.05 were considered significant.

## Results

### Histological examination of decellularization

**Fig. 5:**
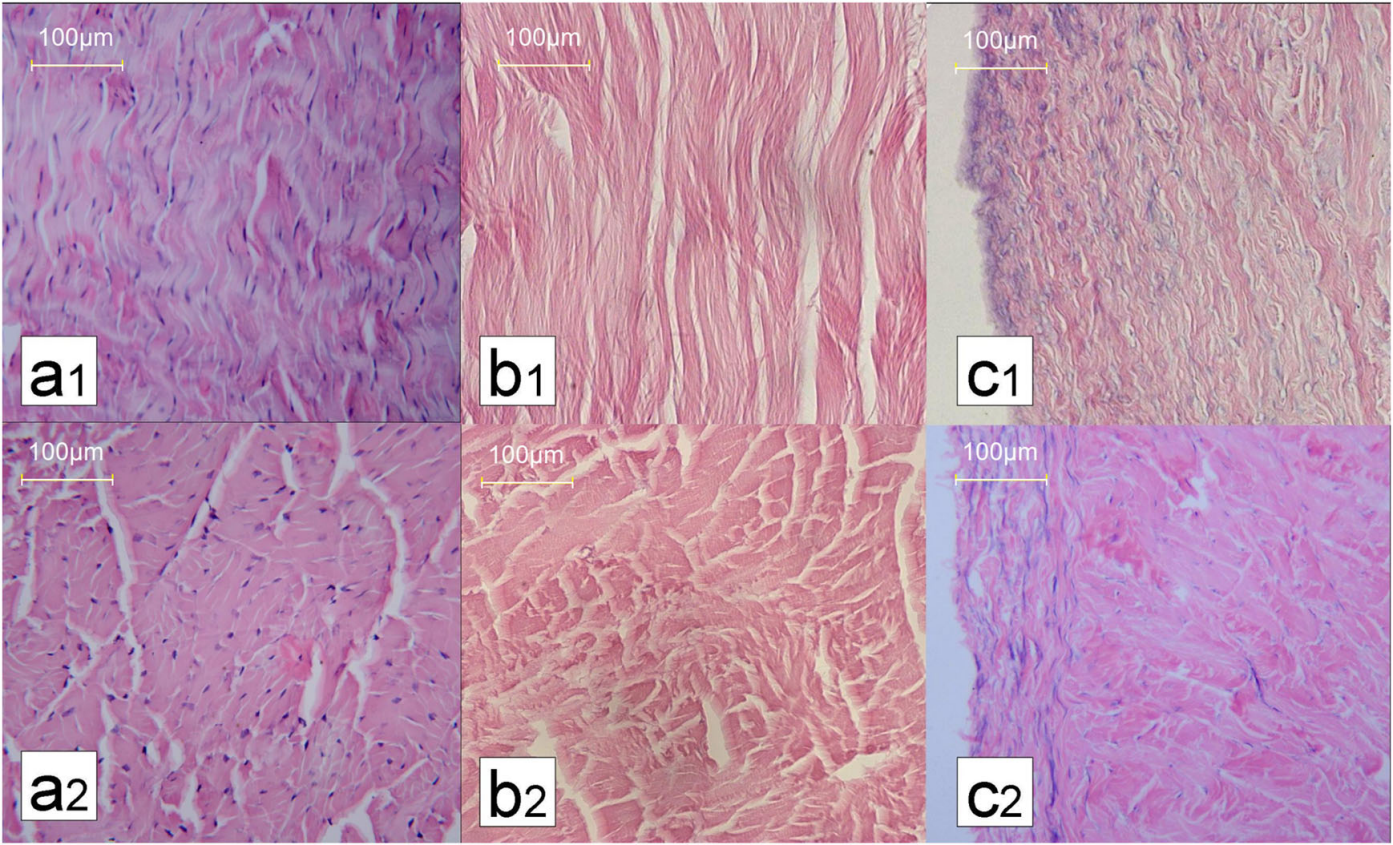
Hematoxylin and eosin (H&E) staining of specimens after decellularization. **a**1, **a**2 Longitudinal sections and cross sections of patella tendons (PT); **b**1, **b**2 longitudinal sections and cross sections of acellular aponeurosis-discission ligament (AADL); **c**1, **c**2 longitudinal sections and cross sections of an intact aponeurosis after decellularization. (magnification ×200)

No nuclear components were detected within the AADL; the collagen structure arrangement did not obviously change (Fig. 5b) compared with that of the normal PT (Fig. 5a), but the interspaces between the collagen fibers were increased after decellularization. In the matrix with the intact aponeurosis treated by the same decellularization procedures, more residual nuclear components accumulated under the aponeurosis (Fig. 5c), as shown by H&E staining. Thus, using the same decellularization procedure, cells in the PT that were removed with aponeurosis discission were more vulnerable than those with an intact aponeurosis.

### Scanning electron microscopy analysis of decellularization

**Fig. 6:**
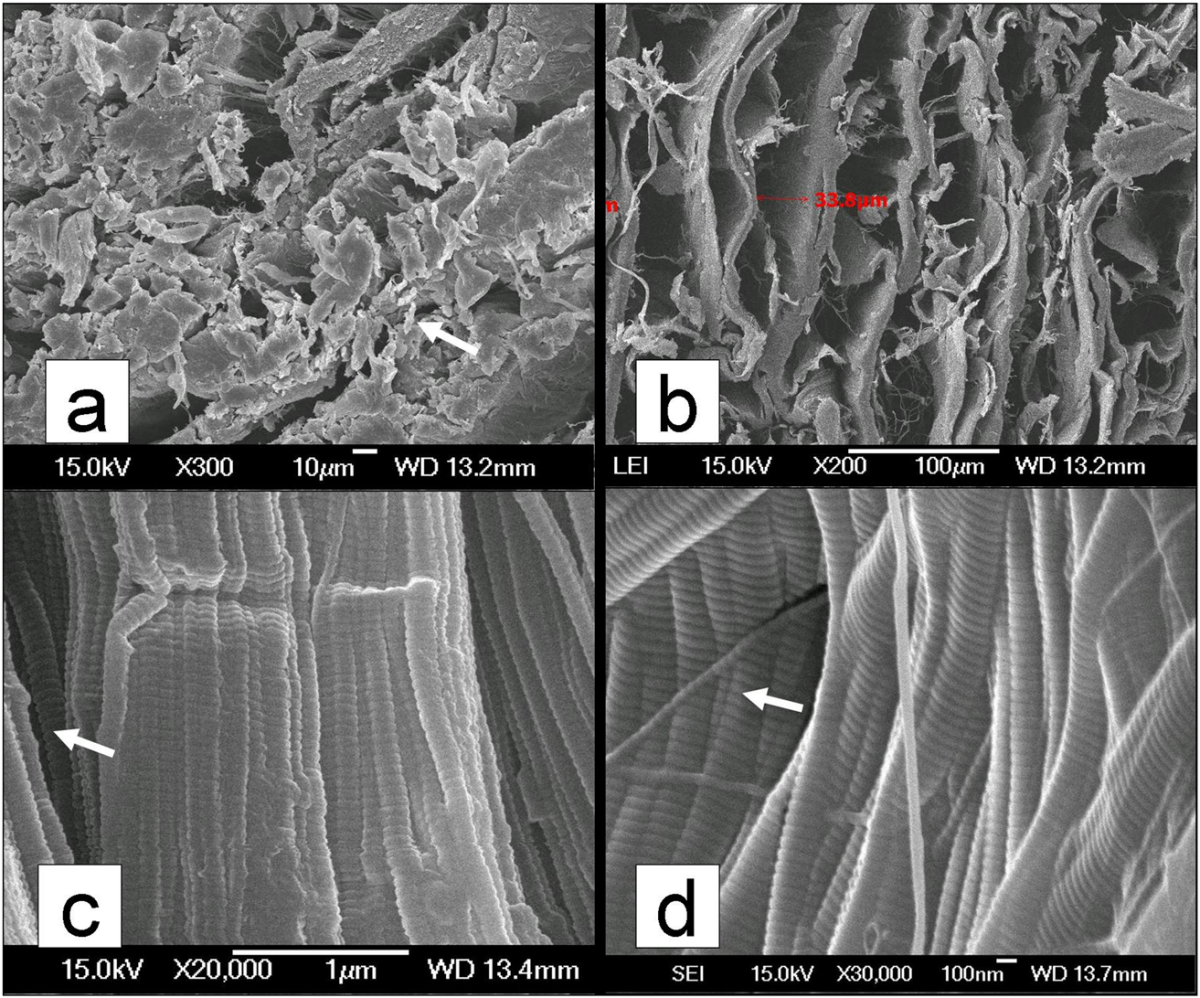
Scanning electron microscopy of specimens after decellularization. **a** Cell debris (white arrow) seen on the cross section of normal freeze-dried patella tendons (PT) (magnification ×300); **b** no cell debris are seen on the acellular aponeurosis-discission ligament (AADL), but the interspaces between the collagen fibers are increased; **c** the normal PT collagen fibrils exhibit a spiral shape (magnification ×20,000); **d** the AADL collagen fibril structure shows no change compared to that of the normal PT (magnification ×30,000)

Electron microscopy examination showed cell debris in freeze-dried normal PT (Fig. 6a); no significant residual cell debris was observed in the AADL, but the interspaces between the collagen fibers had significantly increased (Fig. 6b). The AADL collagen fibril structure (Fig. 6d) showed no change compared to the normal PT (Fig. 6c).

### Determination of DNA content and collagen content

**Fig. 7:**
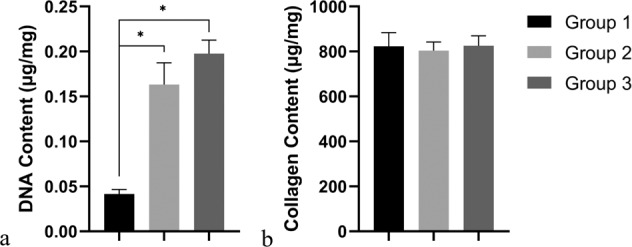
DNA and collagen content of specimens after decellularization. *indicates statistical significance between bars (*p* < 0.05)

The results of DNA content and collagen content tests are shown in Fig. 7. The DNA content of AADL (0.0415 ± 0.005 μg/mg) was significantly lower than that of the natural PT (*p* < 0.05), but that of group 2 (0.1632 ± 0.0243 μg/mg) showed no significant difference compared to the natural PT (0.1977 ± 0.015 μg/mg, *p* > 0.05). The collagen content in specimens of group 1 (822.76 ± 61.26 μg/mg), group 2 (803.90 ± 37.93 μg/mg), and group 3 (825.51 ± 44.43 μg/mg) showed no significant difference (Fig. 7, *p* > 0.05).

### Mechanical testing

**Fig. 8:**
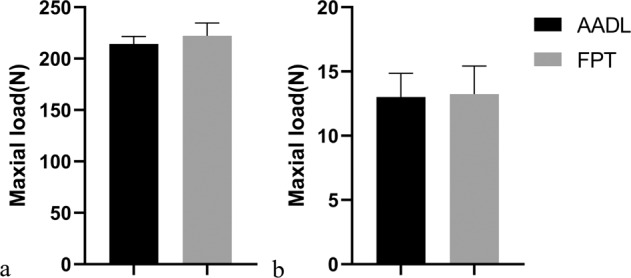
Mechanical testing results of the specimen. No statistical significance was shown between groups (*p* > 0.05)

Mechanical testing of the AADL showed that the maximum load at rupture was 214.33 ± 7.26 N (*n* = 6), and a slight decrease was noted compared to the maximum FPT load, which was 222.17 ± 12.42 N (*n* = 6), but the difference was not statistically significant (*p* > 0.05). No significant difference was observed with regard to the elastic modulus between AADL (13.01 ± 1.84 MPa) and FPT (13.24 ± 2.18 MPa) (Fig. 8, *p* > 0.05).

### In vitro cytocompatibility of the AADL

**Fig. 9:**
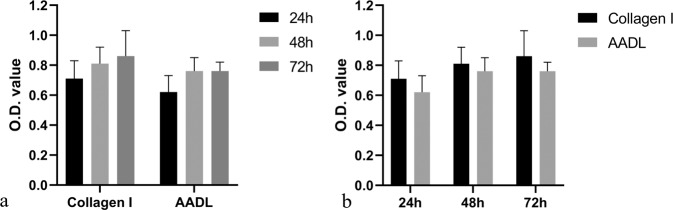
MTT assay for proliferation of cells on specimen surface. No statistical significance was observed between AADL and collagen (**a**, *p* > 0.05) or among the 24, 48, and 72 h time points (**b**, *p* > 0.05)

The colorimetric MTT assays showed that the absorbance at 540 nm of cells on AADL slices was slightly lower than that on collagen coating, but this had no significant effect on the proliferation of cells on either surface (*p* > 0.05) (Fig. 9).

**Fig. 10:**
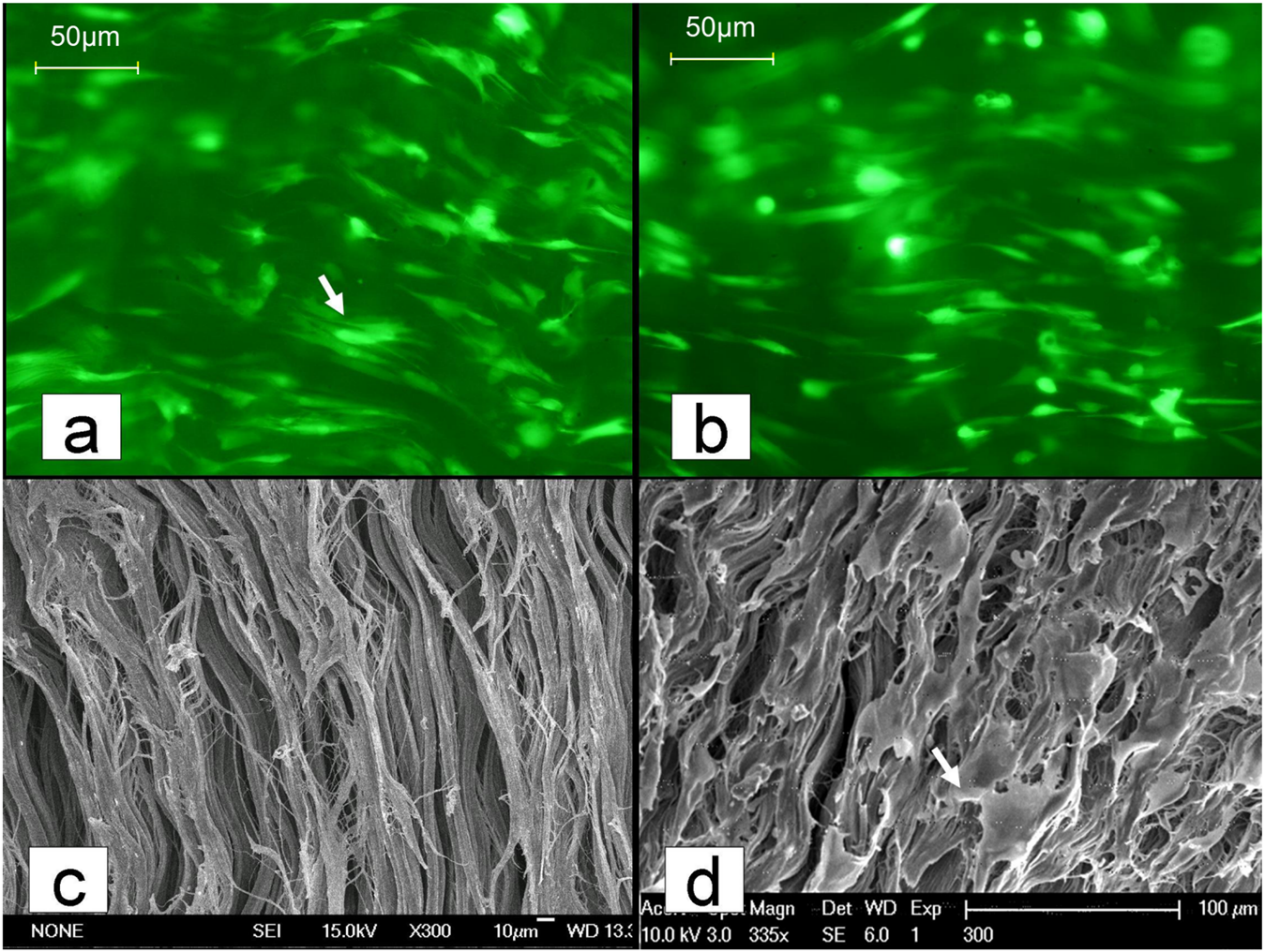
Fluorescence microscopy and scanning electron microscopy of GFP-tagged fibroblasts on specimens. **a** Cocultures at 24 h show more fibroblasts attached to the acellular aponeurosis-discission ligament (AADL) surface (as indicated by the white arrow on the green fluorescent display, magnification ×400); **b** the fibroblast density in cocultures incubated for 72 h did not change significantly (magnification ×400); **c** AADL surface before coculture with cells (magnification ×300); **d** cocultures at 72 h show more seeded cells (indicated by the white arrow) attached to the AADL surface (magnification ×335)

After 24 h of coculturing (Fig. 10a), GFP-tagged fibroblasts were directly observed on AADL slices by fluorescence microscopy. The cells were isolated, and they exhibited longitudinally oriented collagen fibers; moreover, the cell density did not change significantly at 72 h (Fig. 10b). Scanning electron microscopy analysis showed that cells were attached to the AADL collagen fibers (Fig. 10d).

### In vivo host cytocompatibility and histocompatibility

**Fig. 11:**
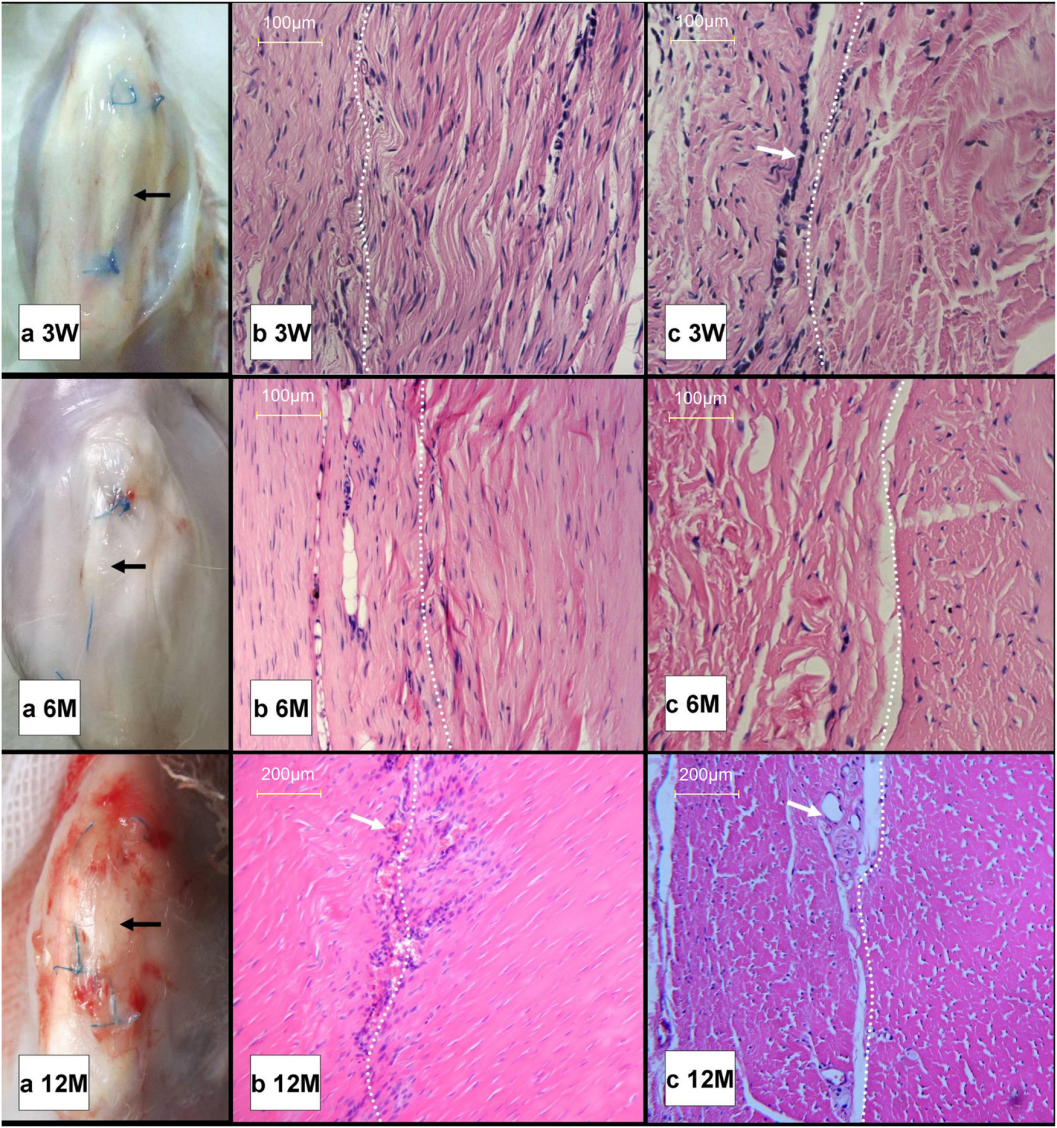
In vivo host cytocompatibility and histocompatibility results. **a** Gross observation at 3 weeks, 6 months, and 12 months after implantation. The implants are indicated by black arrows. **b**, **c** Longitudinal section and cross section of hematoxylin and eosin (H&E) staining (magnification ×200). The right area of the dashed line indicates the AADL, whereas the left area is the host PT. A small monocyte distribution is indicated by the white arrow (**c** 3 W), and angiogenesis is also indicated by the white arrow (**b** 12 M and **c** 12 M; magnification ×100)

From qualitative observation, the rabbits’ wounded knee joints showed good activity; the implants healed well with their surrounding ligaments, and no severe adhesion or significant formation of granulation was found around the AADL after implantation at 3 weeks, 6 months, or 12 months (Fig. 11a). At 3 weeks, the site showed that fibroblast-like cells migrated deep into the AADL and oriented longitudinally along the long axis of the collagen fibers. However, a small number of monocytes were distributed at their interface (Fig. 11c 3 W), and their collagen fibers appeared slightly looser than those of the host PT (Fig. 11b 3 W). At 6 months, the structure of the AADL collagen fibers improved, and the amount of monocyte infiltration decreased (Fig. 11b and 11 6 M). At 12 months, the AADL integrated well with the host PT, whose fibroblast-like cell distribution and collagen fiber structure resembled that of the host PT. In addition, more angiogenesis was found at the healing interface (Fig. 11b and 11 12 M). No significant evidence of infiltration by inflammatory cells, such as multinuclear giant cells, eosinophils, lymphocytes, or macrophages, was observed at any time point.

## Discussion

Most of the current strategies to construct tissue-engineered ligaments have relied on cells seeded into a scaffold. Although the acellular ligament scaffold showed characteristics that were similar to its natural ECM, its porosity was not suitable for seeding cells deep into the tissue in vitro [8, 20, 21]. However, this did not obstruct the migration of fibroblast-like host cells deep into the acellular ligament scaffold in vivo [9]. If the fibroblast-like host cells and other relevant host cells can remold the acellular ligament scaffold with sufficient biomechanical integrity to withstand rehabilitation, the steps of generating and seeding cells into the scaffold may be omitted. Therefore, it is of great interest to identify a method to construct an acellular scaffold that preserves the biological properties of the ECM of the ligament.

The ideal method for the decellularization of ligaments would completely extract the cells while retaining their native structure and biological properties. The study presented here demonstrates that cells are more vulnerable to removal in aponeurosis-discission PT than in tissue that has an intact aponeurosis using the same procedures for decellularization. The dosage of chemical detergents and the decellularization procedure were also reduced compared with those reported by Cartmell [7]. No obvious residual cellular components of the AADL were identified by histological and scanning electron microscopic analyses. Its mean residual DNA content (0.04 μg/mg) was lower than that in commercially available acellular scaffolds, such as Restore™ (Porcine small intestinal submucosa) and TissueMend™ (Bovine fetal dermis) [22], as well as in the acellular ligament scaffold reported by Whitlock [9]. The AADL also had satisfactory mechanical properties and good cytocompatibility in vitro, likely due to its retention of the native ultrastructure of collagen fibrils. The AADL also proved to integrate well with the host ligament in vivo. Histological examination showed that host fibroblast-like cells that migrated deep into the AADL were distributed evenly and oriented longitudinally along the long axis of its collagen fibers. The structure of the AADL collagen fibers was similar to that of the host ligament. In addition, no obvious immune rejection or inflammatory responses were induced by the host PT.

During the decellularization processes, the internal collagen fibers of the aponeurosis-discission ligament are more exposed to the decellularized reagents. Therefore, we should select reagents that have little negative effect on collagen protein. The main function of Triton X-100 is to disrupt lipid–lipid and lipid–protein interactions while leaving protein–protein interactions intact; the functions of the low concentrations of trypsin and EDTA are mainly to disrupt cell adhesion to the ECM, and DNase is used to hydrolyze the DNA components [16]. Therefore, these selected reagents should have no negative effect on the collagen protein [23]. Although the interspaces between collagen fibers in the AADL were increased, no significant changes in its mechanical properties were observed. This is likely due to the continuous structure of the mature ligament/tendon fiber and the fact that force is directly transmitted through the collagen fibers rather than the connection collagen structure [24–28].

Allogeneic/xenograft antigenicity is mainly conferred by the Gal antigen in the cell membrane, DNA, and related proteins [10–13, 29]. The antigenicity of an acellular ligament matrix is primarily due to being largely made up of collagen. Because the collagen structure is conserved across species [30–32], it possesses little inherent immunogenic potential [15, 29]. When used as tissue engineering scaffolds, seeded cells did not easily reseed into the interior of the acellular matrix ligament as determined from in vitro tests [8, 9, 21, 28, 32], but, in vivo, host fibroblast-like cells were still able to migrate into the AADL. In this study, an histological examination revealed that the collagen structure of the acellular ligament matrix gradually improved at 3 weeks, 6 months, and 12 months after implantation. Whether acellular matrix remodeling was related to the migration of fibroblast-like cells into the tissue and how these affect reconstruction and biomechanical changes remains unclear and is an active area of research.

## Summary

Intact aponeurosis of the ligament can hinder the removal of cells during decellularization processes. Using an appropriate aponeurosis discission before decellularization, cell components were extracted more easily with a low dose of detergent in a short time period. This decellularization method showed no obvious impact on collagen fibril structure and no significant adverse effect on mechanical properties. The AADL also exhibited good cytocompatibility and histocompatibility, and the results of the H&E staining revealed that the allogeneic PT defects reconstructed by AADL at 12 months after implantation were satisfactory.
